# The Rehabilitation of Women Who Have Had a Mastectomy

**DOI:** 10.3390/nursrep15040133

**Published:** 2025-04-16

**Authors:** Rogério Ferreira, Carla Jeronimo, Ana Mira, André Pereira, Soraia Serrano, Maria Fatima Marques, Cristina Lavareda Baixinho, César Fonseca, Luis Sousa

**Affiliations:** 1 School of Health, Polytechnic Institute of Beja, 7800-111 Beja, Portugal; carla.jer@live.com.pt (C.J.); anamira49@gmail.com (A.M.); andrefborges01@gmail.com (A.P.); soraia.serrano@hotmail.com (S.S.); 2 Comprehensive Health Research Centre, 7000-811 Évora, Portugal; cfonseca@uevora.pt; 3 Rehabilitation Nursing Department, Nursing School of Lisbon, 1600-190 Lisbon, Portugal; fmarques@esel.pt (M.F.M.); crbaixinho@esel.pt (C.L.B.); 4 Nursing Research, Innovation and Development Center of Lisbon (CIDNUR), 1900-160 Lisbon, Portugal; 5 São João de Deus School of Nursing, University of Évora, 7000-811 Évora, Portugal; 6 Atlântica School of Health, Atlantic University, 2730-036 Barcarena, Portugal

**Keywords:** women who have had a mastectomy, rehabilitation, rehabilitation nursing programs, functionality, quality of life

## Abstract

**Background**: Breast cancer is one of the main causes of mortality among women, and mastectomy has a significant effect on the body image, sexuality and psychology of women. The aim of postmastectomy rehabilitation is to improve functionality, minimize complications and promote well-being and quality of life. **Objectives**: This study aimed to understand the role of nurses specializing in rehabilitation nursing in the rehabilitation of women who have had a mastectomy. **Methods**: This was a qualitative, exploratory and descriptive study. The participants included seven nurses specializing in rehabilitation nursing who provided structured narratives about their experiences and care strategies in the rehabilitation of women who have had a mastectomy. The interviews were analyzed by thematic categorization via content analysis. **Results**: Three main categories emerged: the meaning of care, professional intervention strategies and health gains. Care is seen as a developmental and person-centered experience, with a focus on preventing complications. The interventions prioritize personalized projects, emotional support and self-care training. **Conclusions**: The rehabilitation of women postmastectomy depends on a holistic and individualized approach centered on the person through emotional and functional support. Rehabilitation interventions improve the functionality, quality of life and autonomy of women and are essential for preventing complications and promoting the acceptance of new health conditions.

## 1. Introduction

Among cancers, breast cancer has the highest incidence and mortality rate among women [1]; it has a substantial impact on society, not only because of its high incidence rate and association with severity, but also because it affects an organ that is symbolic of maternity and femininity [2].

Most women with breast cancer undergo local surgical treatment, complemented with radiotherapy; for some women, systemic treatments such as chemotherapy and hormone therapy are needed. The American Cancer Society [3] defines two types of breast cancer surgery: in breast-conserving surgery, only the tumor and a small part of the surrounding tissues are removed, whereas in radical mastectomy, the entire breast is removed, including some nearby tissues and the axillary lymph nodes. Currently, the surgery of choice is modified radical mastectomy, in which the pectoral muscles are preserved [1].

Breast surgery causes body image, sexuality, psychological and social changes in women, creating challenges in daily life [4]. In addition to these changes, the independence and autonomy of women are markedly compromised after mastectomy, with limitations in terms of functionality, potentially hindering or preventing such women from performing some activities of daily living. Among the most frequently highlighted symptoms postmastectomy are reduced range of motion and shoulder muscle strength, chronic pain, sensory disorders and lymphedema, which have enormous impacts on the physical activity and quality of life of women [5].

Rehabilitation after mastectomy should be a continuous process that begins at an early stage, allowing for the planning of a structured and individualized rehabilitation program, valuing health education in the preoperative, postoperative and post discharge periods, and enabling women to provide self-care and respond to future challenges [6]. Rehabilitation plans for women with breast cancer should be appropriate to the stage of treatment in which they are in.

Studies focusing on the evaluation of rehabilitation programs for women who undergo a mastectomy have revealed gains in terms of the functional capacity of the limb on the mastectomy side, with increased range of motion of the shoulder joint, increased muscle strength, and decreased adhesions, seromas and lymphedema, among other complications, as well as increased physical activity, quality of life and self-perceived well-being [1,5,6,7,8,9,10,11,12,13,14,15,16,17,18,19,20,21].

In Portugal, nurse specialists in rehabilitation nursing play a key role in the entire rehabilitation process, with a special contribution to the prevention of complications that may arise and affect the quality of life of women postmastectomy [6].

In addition to technical care, the intervention strategies of these nurse specialists include a humanized approach centered on women and their needs, with support in transition processes [22,23] and health education strategies [23,24] and by implementing motor and cardiorespiratory training programs, in addition to training programs for activities of daily living, aiming at their independence, autonomy and adaptation to mobility limitations [9,10,11,12,13,14].

Nursing plays an important role in the entire health care delivery process, from the needs assessment phase to the planning of responses appropriate to the needs of individuals, the effective provision of care and the evaluation of the results of interventions, as well as the ability to reformulate plans of action. Nurse specialists in rehabilitation nursing, due to their knowledge and acquisition of skills in the field of rehabilitation, have the ability to design, implement and monitor specialized care plans aimed at addressing a set of real or potential problems that women face in the period after mastectomy, always seeking to promote, recover or maintain functional capacities, prevent complications and avoid or minimize the impact of disabilities that arise from this process. We conducted a study to better understand the rehabilitation process from the perspective of these special health care providers. In view of the above, our objective was to understand the perspective of nurses specializing in rehabilitation nursing in relation to the rehabilitation of women who have had a mastectomy.

## 2. Materials and Methods

This was a qualitative, exploratory and descriptive study. According to Colorafi and Evans [25], such studies are based on the general principles of naturalistic research and allow for the development of knowledge in health, because qualitative research can help in understanding complex phenomena by investigating the experiences, beliefs, behaviors, attitudes and interactions of people.

The inclusion criterion for the participants was rehabilitation nurses with experience in providing rehabilitation care to women postmastectomy in the immediate and late postoperative periods. The researchers used the contact networks of these professionals to identify subsequent participants, and initial contact by telephone was employed to verify whether individuals met the criterion. We considered nurse managers without direct involvement in the planning and implementation of rehabilitation programs as an exclusion criterion. Despite the difficulty in obtaining participants who were systematically involved in the rehabilitation care of women who underwent mastectomy, seven (7) rehabilitation nurses from various health institutions in Portugal participated in this study. The number of subjects was determined by the data saturation criterion.

Narrative as a data collection procedure most commonly used in social and health research, was chosen with the purpose of collecting narratives about the meanings of lived experiences [26] in the context of clinical practice by nurse specialists in rehabilitation nursing. The guide for narratives was structured; to ensure the content validity of the instrument, the guide was initially reviewed and approved by two judges.

During the process, the main intention was to ensure that the guiding questions of the narrative were framed within the defined objectives and allowed for the reorganization of the experiences of caring for women postmastectomy in a coherent and meaningful way, giving meaning to the experience and providing a narrative that integrated clinical practice [26].

The guiding script for the narrative aimed to provoke reflection on and analysis of specialized rehabilitation care for women postmastectomy from the perspective of rehabilitation nurses. The script included five questions.

On the basis of the care processes in which you intervened as a nurse in the rehabilitation of women postmastectomy, I would like to obtain your responses to the following questions:

- How was the experience of caring for women who have had a mastectomy? What meaning do you attribute to this experience of care?
- What are the strategies used in the rehabilitation of women who have had a mastectomy?
- Which rehabilitation nursing interventions do you consider to be a priority in the care of women who have had a mastectomy?
- If you had to design a multicomponent rehabilitation nursing intervention for women postmastectomy, what dimensions and interventions would you recommend?
- What are the health gains resulting from the implementation of these interventions?

In compliance with the ethical principles that should guide the preparation of a study of this nature, a request was made to the ethics committee at an institution of higher education, and contact was established with the Nurses Specialists in Rehabilitation Nursing. At the institutional level, this study (Protocol No. 3/2024) received a favorable opinion from the Ethics Committee of the Polytechnic Institute of Beja on 27 February 2024. The nurses, as participants, were assured that their participation in this study would be strictly voluntary with the possibility of withdrawing at any time, without having to justify their decision and without any effects on future treatment that concerns them. The anonymity and confidentiality of the data provided by the participants were guaranteed, considering professional secrecy as an obligation and duty. In addition, the participants signed an informed consent form.

Contact with the participants was made by all the researchers at two time points. First, the participants were contacted by telephone to validate their interest in participating in this study and their compliance with the predefined inclusion criteria. Interested participants were sent an interview guide and provided informed consent via e-mail.

All the participating nurses prepared their narratives, providing the researchers with noneditable responses, as well as informed consent, which was duly signed by the nurses.

Data processing was operationalized by the principal investigator with the objective of standardizing the procedure. The confidentiality and anonymity of the data obtained and the identities of the participants were ensured throughout the process. The data were stored by the principal investigator in a specific encrypted device with the plan of restricting access to people outside the study for a period of 5 (five) years.

The names of the participants are replaced by identification numbers (P1, P2, P3, …) when used either in the responses or in publications. In the assertions (registration units) presented in the study results, measures were adopted to protect the identity of the participants. Data protection extended from the selection of participants to the collection, analysis and dissemination of study results.

Narratives, as a research technique, are a primary source of reflection developed by study participants, especially in the fields of education and research [26]. The results obtained from the narratives were analyzed using the content analysis technique and thematic category analysis [27]. In the first stage, a general reading was carried out, with the purpose of verifying whether the information collected was related to the objectives of this study. Therefore, the narratives received constituted the focus of analysis, which corresponds with the material analyzed and constitutes the corpus of the research [27]. In a second stage, we proceeded to explore the material. We based ourselves on three essential procedures: the choice of analysis units, enumeration and categorization [27]. We defined the units of analysis as recording units and context units. We considered the theme as the unit of registration, which, according to what Bardin [27] defends, would be a statement about a subject, to which a vast set of singular formulations can be affected. We defined the context unit as the unit of understanding to encode and understand the exact meaning of each recording unit, that is, each participant’s response to the guiding questions of the narrative. In the enumeration, we did not attribute importance to the frequency of the recording units, but rather to the presence of elements in the subjects’ narratives. In categorization, the categories were defined, supported by the theorization of the object of study and the definition of the objectives associated with it. We sought to ensure the quality of the categorization process with exclusivity, homogeneity, relevance, objectivity and productivity [27].

The analysis ensured the inference and interpretation of the data. Through semantic analysis, we sought to understand the specific meaning of statements, i.e., recording units. This inferential process was structured by defining the indicators defined for each category [27] and understanding the meaning that emerges from the narratives of these participants in this study.

Several procedures were developed to ensure the quality of the research:

- The study protocol was followed, with a rigorous description of the procedures from planning to collecting data, reporting personal assumptions and providing potential limitations [25];
- Explanations of the procedures for data collection and analysis, as well as the theoretical framework and justification of the study, were provided;
- A review by peers/judges—the collaboration of two judges/experts on the subject—was requested; these individuals validated the content analysis and made suggestions for improvements;
- Constant discussions regarding the findings and the coding process among the team made it possible to ensure the transparency of the work. The team had experience in conducting qualitative studies;
- In the definition of the categories, objectivity, exhaustiveness, representativeness, homogeneity, exclusivity and pertinence were ensured [28].

## 3. Results

This study included seven nurses with postgraduate training in nursing. All participants were nurses specializing in one of the areas defined by the order of Portuguese nurses, i.e., the area of rehabilitation nursing. In this sample, all participants were female. They worked in Algarve (2), Évora (1) and Lisbon (4), in different care contexts, namely in hospital inpatient services (4), home care (2) and community contexts (1).

This study comprises three categories: “meaning attributed to rehabilitation care for women who have had a mastectomy”, “professional intervention strategies” and “health gains arising from the implementation of care strategies”. Table 1 presents the categories and indicators that emerged in this analysis.

### 3.1. Meaning Attributed to Rehabilitation Care for Women Who Have Had a Mastectomy

This category encompasses indicators such as developmental experience, person-centered experience and preventive experience.

**Development experience**

The meaning attributed to rehabilitation care for women who have had a mastectomy is a developmental experience due to the importance that the provision of care has in demystifying prejudices regarding the bodies of women by contributing to improvement in autonomy and in the adaptation of women to a new reality. In addition, there is an impact on nurses with regard to rethinking their life course and redefining their priorities.

*The mastectomized woman experiences a process of loss and grief in the face of her body and the image that defines her as a woman. In addition, the way I have witnessed and accompanied countless women dealing with this process has been, I dare say, inspiring. More than that, it raises awareness, I would say! Insofar as it has forced me to demystify prejudices regarding women’s bodies, to redefine priorities in all my self-care and to rethink even my life purpose/path.*

(P2)

*The fact that I provide care in the service […] allows me to be one of the first actors in the process of rehabilitation of the mastectomized woman at home, in her adaptation to the home context at a very early stage of her adaptation process to the limitations associated with the intervention (most women go home at a very early stage of their postsurgical process, still with vacuum drainage systems in place and with many movement limitations—both in amplitude, strength, sensitivity and pain). I consider it to be an extremely significant experience both for the women and for me as a rehabilitation nursing professional, as it enhances the improvement in these women’s autonomy and is a crucial factor in their adaptation to their new reality.*

(P3)

**Person-centered experience**

For one of the participants, the care experience should be centered on the person and their family, valuing a holistic intervention to the multiple dimensions of care.

*Mastectomy has a great impact on women, not only because of the associated health-disease process but also because the breast has great symbolism in a person’s self-image and sexuality. A mastectomy is often perceived by a woman herself as a mutilation, which causes changes in self-esteem, transforming the way the woman sees herself and her relationship with others. Throughout the experience of care, I observed the importance of providing care centered on the person and family, intervening in all its dimensions, because there is no point in intervening in physical rehabilitation without psychological intervention. If the intervention plan does not make sense to the woman, she will not adhere to it.*

(P5)

**Preventive experience**

Experience with care was a determinant in the prevention of complications resulting from surgery. The statement that follows reflects the meaning attributed by one of the participants.

*As a nurse specializing in rehabilitation care, I have to consider that there are three moments in the care of women with mastectomies: the preoperative period (phase 1), the postoperative period (phase 2) and the period of sequelae or chronicity (phase 3). Whenever possible, an approach should be performed before surgery to prepare the patient for the next period. The institution of an early rehabilitation program can prevent decreased range of motion, decrease the volume of edema ipsilateral to surgery, and improve issues related to pain and functional disability inherent to limitations.*

(P4)

### 3.2. Professional Intervention Strategies

The following categories are considered in this category: project development, support in transition processes, self-care training, health education, comfort care, evaluations of functionality and health status, the implementation of motor and cardiorespiratory training programs and the implementation of programs for ADL training.

**Project development**

For the participants, the development of projects is a professional development strategy to be implemented among women postmastectomy. The rehabilitation nursing consultation and rehabilitation programs adapted according to the family, social and work context are the object of this strategy. This indicator was highlighted by two rehabilitation nurses.

*I developed a project that is being approved by the nursing directorate of (…) entitled ’Rehabilitation nursing consultation for people undergoing breast surgery—mastectomy’.*

(P1)

*The strategies involve creating a rehabilitation program adapted and personalized to each patient and directed to the reality of that patient, considering the social, family, personal and work context. The success of a rehabilitation program depends on these variables.*

(P4)

**Support in transition processes**

Support in the transition process, by providing the woman with emotional support, managing their emotions and offering positive reinforcement, are essential to help her deal with her health situation and body image changes. This indicator was highlighted by four rehabilitation nurses.

*Support in the acceptance of self-image (…)*.

(P1)

*(…) without neglecting interventions in the psychological and social spheres of women, in the process of adapting to the disease and changes in body image.*

(P6)

*Furthermore, changes in self-image trigger the implementation of interventions, with the progressive use of mirrors and approaches in the field of sexuality.*

(P6)

*All the follow-up of women involves an enormous component of emotional support, the management of emotions and positive reinforcement, and valuing and praising the importance of their involvement in their own recovery process.*

(P2)

*Emotional support (…) is a fundamental and essential component of my intervention throughout a woman’s hospitalization. It became evident throughout my experience in caring for women with mastectomies that encouraging the expression of emotions/feelings and the clarification of doubts combined with the education, empowerment and training of women led to a faster return of independence and satisfaction with their breastfeeding and daily life activities.*

(P2)

**Self-care training**

For the participants, self-care training is a key strategy in the rehabilitation of women with mastectomies, benefitting their functional independence.

*I try to provide them with some active exercises that allow them, at home, to somehow maintain limb activity and movement and that promote their self-care training.*

(P1)

*(…) the empowerment of the person with participation restriction for their self-care, the maximization of the function of the limb homolateral to the mastectomy, promoting independence.*

(P1)

*Intervention in self-care training, maximizing the functional potential of women and making them as independent as possible, is also a priority.*

(P7)

**Health education**

Health education strategies, whether implemented individually or in groups, are essential for women to address the surgical process, their health situation and the development of their activities of daily living due to the changes resulting from mastectomy.

*In the service where I work, all the women who undergo surgery are admitted the day before surgery. This first day is described by all of them as a day of great anxiety and fear in the face of the unknown. As such, one of the strategies used has been group sessions of health education and training on the interventions preoperatively that will be developed in the postoperative period, with the women grouped according to the type of surgery to be performed. These sessions allow the reception of women at the service, the provision of information directly related to the surgery (confirmation of the laterality of the surgery, operative time, ward-operating block circuit, estimated average for days of hospitalization, location of sutures and/or drains and the respective care to be taken), the provision of information regarding the preoperative routines, and the clarification of doubts in general.*

(P2)

*As a rehabilitation nurse in the care of women with mastectomy, I consider interventions (…) of health education with regard to care in the different activities of life (hygiene, clothing and adornments, domestic activities, work, sports and leisure activities to be a priority) for the prevention of lymphedema.*

(P2)

*Teaching about self-mobilization and strategies to overcome the limitations resulting from mastectomy.*

(P5)

**Comfort care**

Comfort care was highlighted by four of the rehabilitation nurses. Interventions for pain control, correct positioning and lymphatic drainage massage are notable in the participants’ narratives.

*(…) activities were developed aimed at comfort and at the needs that each woman identified.*

(P7)

*In the postoperative period, pain control and correct positioning are priorities (…).*

(P6)

*The early institution of lymphatic drainage massage should be considered, aiming at decreasing the amount of drained fluid, improving lymphatic reabsorption, and preventing or reducing lymphedema.*

(P4)

**Assessments of functionality and health status**

An evaluation of the range of motion of the limb homolateral to the mastectomy and various aspects related to health status, such as pain, surgical wounds, and the presence of drains, were highlighted by the rehabilitation nurses in the evaluation of functionality and health status. This indicator is highlighted in the narratives of three rehabilitation nurses.

*For the rehabilitation process of women with mastectomies, it is essential to first understand what knowledge the women already have regarding their new physical condition; assess their behavior/acceptance regarding their body image; assess pain; assess movement tolerance; and evaluate the range of motion of the homolateral side to the mastectomy (…).*

(P3)

*To ensure safety, it is important to monitor pain before, during and after the intervention; care to be taken while the drain is still available and the surgical wound has not yet closed; monitoring the range of motion (according to tolerance) and preventing joint stiffness; and monitoring the swelling of the UL ipsilateral to surgery (to prevent lymphedema).*

(P5)

**Implementation of motor and cardiorespiratory training programs**

Motor and cardiorespiratory training programs involve rehabilitation nursing interventions adjusted to the health situation of each individual. Motor and cardiorespiratory training interventions must be interconnected, especially motor function re-education during the immediate postoperative period with the mobilization of the ipsilateral limb. Teaching, instructing and training interventions for positioning and muscle and joint movement are initiated early and are essential to promote the self-care of women postmastectomy and contribute to their functional recovery. This indicator was addressed by all participants, indicating its relevance in rehabilitation nursing care.

*In the postoperative period, an individualized intervention strategy is chosen, with daily sessions with each woman individually whenever possible. Allied to the motor and respiratory functional reeducation interventions that are developed in each session (…).*

(P2)

*(…) in the immediate postoperative period, Rehabilitation Nursing interventions should include positioning in the bed contralateral to the mastectomy, relaxation and stretching exercises of the cervical region and shoulder girdle to relieve pain and muscle contraction, active-assisted low-amplitude exercises with the upper limbs (shoulder flexion, abduction and rotation), and respiratory kinesitherapy (with costal opening, taking into account the low joint movement amplitude).*

(P4)

*During hospitalization, the interventions I develop in terms of cardiorespiratory functional reeducation and functional motor reeducation are as follows (beginning in the preoperative period): Instruction on the positioning of rest and relaxation; Instruction and training of control and dissociation of respiratory times; Instruction and training of abdomino-diaphragmatic breathing; Encouragement of active mobilization in bed during the period of bed rest; Instruction and training on the lifting technique (for the side opposite to the surgery); Instruction and training on the postural correction technique; Instruction and training on cervical relaxation and mobilization exercises, scapulohumeral mobilization and relaxation and shoulder girdle mobilization; Instruction and training on the handgrip exercise; Instruction and training on active mobilization of the upper extremities (elbow flexion/extension; flexion/extension, cubital deviation and radial deviation of the wrist; flexion/extension, opposition, adduction and abduction of the fingers); and Instruction and training on exercises for lifting the upper limbs up to the shoulder line (90°) with dissociation of respiratory times (inspiration when flexing the shoulder and expiration when returning to the point of origin).*

(P2)

*When a woman removes the suction drains (thoracic and/or axillary), the following interventions are added: scapulohumeral abduction exercise (wall climb); pendulum exercise; Pulley exercise (using the door with a sheet); and mobilization exercise with a towel. As a complement to the previously identified interventions, an abductor shoulder pillow is provided for periods of rest (…) and its purpose and correct use are explained.*

(P2)

*The woman is instructed to perform the exercises 3 times a day, progressively increasing the number of repetitions, starting with 5 repetitions up to a maximum of 15.*

(P2)

*In the care of women with mastectomies, the inclusion of the psychological and physical dimensions of a Rehabilitation Nursing program is fundamental in the design of a Rehabilitation Nursing program. This reality implies the definition of a multicomponent program that includes correct positioning in bed, contralateral to the mastectomy, positioning of the ipsilateral limb, relaxation and stretching exercises to relieve pain and muscle contraction, and low-amplitude active-assisted exercises.*

(P6)

**Implementation of an ADL training program**

The implementation of training programs for activities of daily living is one of the competencies of nurses specializing in rehabilitation nursing. The purpose of these intervention strategies is to adapt women who have had a mastectomy to mobility limitations and maximize their independence and autonomy, thus ensuring a better quality of life. This indicator was addressed by three of the participants and expressed in the following statements.

*Reeducation techniques in ADLs adapted to the reality of each patient.*

(P4)

*The teaching and training of self-care, particularly in hygiene and dressing and undressing, in the adaptation of the bra and eventual adaptation of external prostheses.*

(P6)

*Self-care training (use of clothing that is loose on the upper part, preferably open at the front; first, dress the arm homolateral to the surgery and then the healthy arm, and undress in reverse).*

(P5)

### 3.3. Health Gains Arising from the Implementation of Care Strategies

This category encompasses the following indicators: recovery of functionality, autonomy, acceptance of the health situation, decreased incidence of complications, improved quality of life and decreased length of hospital stay.

**Functional recovery**

The nurses believed that the strategies they use with women who have had a mastectomy are crucial in the recovery of functionality. The following statements express this type of gain.

*(…) results in the restoration of functionality through the recovery of range of motion, prevention of postural changes, prevention of muscle atrophy, prevention of lymphedema, and the reduction/improvement in postoperative complications (such as pain, paresthesia, edema, joint stiffness, functional limitation and joint adhesion).*

(P2)

*(…) the gains in health are related to the promotion of independence (…).*

(P2)

*Gains in range of motion on the homolateral side to the mastectomy, promotion of self-care.*

(P3)

**Autonomy**

Autonomy is one of the gains of the strategies implemented by rehabilitation nurses for women who have undergone a mastectomy. This arises from training processes that help women manage the disease process, overcome difficulties and adjust to their new health condition. The statements that follow express this gain in health.

*(…) the gains in health are related to the (…) autonomy in self-care.*

(P2)

*The health gains resulting from the implementation of these interventions include greater autonomy among women undergoing mastectomy.*

(P3)

**Acceptance of the health situation**

Strategies to support the transition process are essential for women to accept their health situation, which is evident in their satisfaction with and motivation for the rehabilitation process. The statements that follow express these health gains.

*Improved acceptance of women’s new physical condition (promotion of self-image).*

(P3)

*(…) improved satisfaction with self-care, with consequent improvement in quality of life and satisfaction/motivation for the recovery process; better level of adherence to the rehabilitation program.*

(P5)

**Fewer complications**

The reduction in complications resulting from the surgical process results from the interventions of nurse specialists in rehabilitation nursing. These gains are expressed in extracts from the narratives.

*(…) decrease in the occurrence of seromas.*

(P3)

*Decreased occurrence of risks associated with surgery (pain, decreased muscle strength, osteoarticular stiffness, lymphedema).*

(P5)

**Improved quality of life**

The improvement in quality of life is one of the health gains as a result of rehabilitation nursing interventions for women postmastectomy. Rehabilitation strategies, including comfort care, were determinant and are highlighted in the following statements.

*In my opinion, improving the patient’s quality of life will already be a gain.*

(P4)

*Gains related to physical and psychological comfort.*

(P7)

**Decreased hospital stay**

A decrease in hospitalization time is one of the gains highlighted by the rehabilitation nurses. The following statement reflects these gains.

*Decrease in the average length of hospital stay of women with mastectomies.*

(P3)

## 4. Discussion

For the participants, rehabilitation care for women postmastectomy is seen as a developmental experience, a person-centered experience and a preventive experience.

Caring for women postmastectomy is a developmental experience due to the importance of rehabilitation nursing care in helping to demystify prejudices regarding the bodies of women and in adapting women to a new reality. Women experience emotional instability, with feelings of uncertainty and insecurity regarding their health situation. Faced with this experience, nurses should promote the emotional security and autonomy of women postmastectomy, helping them and their family members face the disease process [23]. These moments of interaction are fundamental in valuing individuals in the face of the challenges they experience, promoting their individual growth and stimulating self-care [23], in addition to having an impact on the development of nurses and helping nurses reflect on their life path.

The person-centered care experience considers the uniqueness of each person and their family, valuing a holistic intervention from multiple dimensions of care. In this sense, nurses must respond to the care requirements of women postmastectomy, supported by a philosophy of humanized and welcoming care [23]. The meaning attributed by nurses presupposes the understanding of the perception that women have of themselves and their body image [23].

The experience of person-centered care, from the perspective of the rehabilitation nurses who participated in this study, requires establishing a trusting relationship with women who have had a mastectomy, providing the necessary support with information appropriate to her care situation and emotional needs, and helping her manage feelings of uncertainty and the decision-making process [8,29]. Communication skills are essential to resolve concerns related to body image and in the management of the rehabilitation process, with benefits that empower the women [8,29].

This perspective of care fits the principles presented by Healthcare Global [30] and assumes that each woman is the source of control, and that care is centered on her needs and choices. Family and friends are considered essential parts of the care team for women postmastectomy, and the care environment should be one of comfort and support and conducive to the rehabilitation process. The safety of each woman and her family is a priority of care in the rehabilitation process, and knowledge and information must be shared between each woman and the stakeholders in this process.

The experience of care is considered preventive because of its importance in the prevention of complications, such as decreased range of motion, decreased ipsilateral edema after surgery, pain and functional disability associated with limitations. Rehabilitation nurses have skills that can help women who have had a mastectomy and their families to develop preventive strategies for physical complications that favor rehabilitation [6].

For the participants, the development of projects is a professional development strategy to be implemented among women postmastectomy. Rehabilitation nursing consultation and rehabilitation programs adapted to the family, social and work contexts are the object of this strategy.

Support in the transition process, by providing women with emotional support, help in the management of emotions and positive reinforcement, is crucial to the rehabilitation process. Given the disfigurement of the breast and the change in body perception resulting from mastectomy, it is essential for nurses to provide emotional support that helps women postmastectomy understand and accept body image changes. Since the breast represents women’s femininity, sexuality and fertility, breast mutilation has a negative effect on body image, leading women to report feelings of fear, anger and changes in interpersonal relationships [6]. This is a dimension of care in which multidisciplinary intervention is essential in the rehabilitation process [23]. It is necessary for women to have a support network to help them face these challenges and to resume social contact and activities, which are crucial for improving self-esteem and self-confidence [22,23].

This type of strategy presupposes the involvement of family and friends, as people who women postmastectomy trust, given the importance they assume in her comfort and protection, helping her in the process of accepting her health situation [8,23].

Self-care training is a fundamental strategy in the rehabilitation of women postmastectomy that promotes functional independence.

The implementation of such strategies can help women and their families assume their own health care and activities of daily living, with greater independence and autonomy. These strategies presuppose the definition of goals, teaching self-care, and valuing the uniqueness of each woman, with her doubts and fears, with the aim of increasing her involvement in care and participation in activities of daily living, thus promoting her personal growth [24].

Health education strategies are implemented individually or in groups; they are essential for women who undergo mastectomy to deal with the surgical process and with her health situation and participate in the development of her activities of daily living due to changes that result from mastectomy. Such strategies aim to promote the independence and autonomy of these women, informing them of the care required after mastectomy, which includes care for the ipsilateral upper limb and the recovery of arm and shoulder functionality; the avoidance of sun exposure, burns, scratches and cuts; and not receiving injections, vaccines or blood withdrawals from the ipsilateral upper limb [23]. The information should value the individuality of each person in their learning and rehabilitation process without ignoring the importance of the family in the rehabilitation of women. Health education strategies require the creation of opportunities to be with the woman and caregivers, paying attention to their information needs and their learning style and clarifying doubts, in addition to providing guidance in the care and rehabilitation process [6,23].

The education of women who have had a mastectomy should emphasize rehabilitation programs involving motor and respiratory functional re-education as fundamental strategies for the rehabilitation process [8]. In a randomized clinical trial with a pre- and postdesign that evaluated the effect of a therapeutic exercise educational program on the quality of life and functional capacity of women who have had a mastectomy, it was found that two to four weeks after surgery, compared with women in the control group, women in the intervention group showed significant improvements in shoulder range of motion (flexion, extension and abduction). Quality of life, that is, physical, psychological, social and spiritual well-being, was better in women in the intervention group than in those in the control group two to four weeks after surgery [1].

Comfort care was highlighted by the participants in this study. Interventions focused on pain control, positioning and lymphatic drainage massage are notable in the narratives of rehabilitation nurses.

A reduction in anxiety is also a concern of nurses after surgery. Learning and using relaxation techniques, deep breathing exercises, music therapy, meditation and yoga are essential for women postmastectomy to manage the anxiety and feelings associated with body image changes [8].

The evaluation of the range of motion of the homolateral side to the mastectomy and various aspects related to the health situation, such as pain, surgical wounds, the presence of drains, and swelling of the limb homolateral to the surgery, were highlighted by the rehabilitation nurses in the evaluation of functionality and health status. These findings are in line with those of some authors [7], who argue that given the severity of the adverse effects resulting from surgery, it is essential to implement preoperative interventions, also known as prehabilitation, to improve physical function before and after surgery, to shorten the hospital stay and to have fewer complications during the postoperative period. It is logical that the implementation of these strategies presupposes an adequate evaluation of the functionality and health situation of each woman, and that such strategies yield enormous gains in the rehabilitation process.

The rehabilitation plan of women with breast cancer should be appropriate for the stage of treatment in which they are in and should include a set of adapted and individualized exercises, complemented with health education sessions. The combination of these intervention strategies has a significant effect on the lives of women postmastectomy, decreasing comorbidities and pain associated with mobilization and increasing their quality of life [5].

Motor and cardiorespiratory training programs involve rehabilitation nursing interventions adjusted to the health situation of each woman postmastectomy. As a result of the surgery, the women report pain, decreased range of motion in the homolateral shoulder, decreased muscle strength, impaired mobility of the limb, and fear of moving the limb [9,11]. In view of these complications, motor and cardiorespiratory training interventions are essential in the rehabilitation process, especially motor function re-education after the immediate postoperative period, with the performance of a program involving gradual functional exercises aimed at the functional recovery of the upper limb and shoulder joints and the prevention of complications [10,11].

Functional motor re-education programs should be instituted in the first six months after breast surgery to promote the functional rehabilitation of the upper limbs [12]. This investigation revealed several benefits of different programs involving regular physical exercise, namely, reducing the volume of lymphedema, improving lymphatic circulation, decreasing the risk of lymphedema, reducing pain, increasing muscular strength, increasing range of motion and the function of the upper limbs, improving cardiovascular function, reducing body weight and improving quality of life [10,12,13,14].

In a systematic review aimed at evaluating the effects of physical exercise programs on postoperative shoulder mobility and upper-limb function in people with breast cancer, a meta-analysis confirmed that motor re-education programs significantly improve shoulder flexion and abduction and improve upper-limb function. Additionally, exercise programs, when performed ≤3 times a week or over 8–12 weeks, are more effective at improving shoulder flexion, whereas shorter durations (<8 weeks) and similar frequencies lead to more favorable results in shoulder abduction. Furthermore, resistance exercises when started early (<2 weeks after surgery) result in significant gains in upper-limb function [15].

Another meta-analysis revealed that aerobic and strength training was the most effective intervention, significantly improving quality of life after 12 weeks of intervention [31].

In a randomized clinical trial conducted in Taiwan, the intervention group participated in a 12-week upper-limb rehabilitation program that involved face-to-face education on upper-limb rehabilitation and monthly assessments of upper-limb activities. Controls received standard nursing care. Compared with the control group, the intervention group showed greater improvements in function and symptom levels after the intervention. The quality of life in both groups gradually increased during the study period [16].

In a study by Hou et al. [17], with the objective of verifying the effect of a combination of functional exercises and psychological interventions in postoperative rehabilitation and in combination with interventions for women with breast cancer, functional exercises combined with psychological interventions improved the adherence of women to treatment, as well as their psychological status, shoulder range of motion and quality of life.

Many women have difficulties and need help performing basic and instrumental activities of daily living, such as personal hygiene, walking, transfers, household activities, shopping and transportation, after mastectomy [9]; therefore, intervention strategies are fundamental in promoting the adaptation of women postmastectomy to the limitations of mobility and the maximization of their independence and autonomy.

A quasiexperimental study by Rodrigues et al. [9] aimed to evaluate the effectiveness of a rehabilitation nursing program in improving the self-care performance of women undergoing breast surgery with axillary lymph node dissection. A home rehabilitation program for 12 weeks led to improved functionality of the upper limb ipsilateral to surgery, with an influence on the ability of these women to perform self-care tasks, such as washing and drying their hair, washing their backs and putting on a pullover.

Exercise programs after surgery have a beneficial effect on the range of motion of the homolateral shoulder [14] and, inherently, on the functional capacity of women postmastectomy. In a study by Majed et al. [1], there was an improvement in shoulder flexion, extension and abduction as a result of an exercise program performed by the participants.

A quasiexperimental study in which a three-month rehabilitation nursing home program was implemented revealed that the functionality of the upper limb ipsilateral to surgery improved significantly after the program [9].

Autonomy is the ability to control and be responsible for actions and decision-making, in accordance with the context [32]. This is one of the gains of the strategies implemented by rehabilitation nurses for women who have undergone a mastectomy. This autonomy arises from training processes that help women manage the disease process, overcome difficulties and adjust to their new health conditions. These findings agree with the view of Merêncio and Ventura [6], who suggest that interventions implemented by rehabilitation nurses are aligned with a process of empowering women and caregivers, which promotes autonomy and independence through the definition and implementation of a set of strategies to help them in the transition process and in their well-being.

A randomized clinical trial by Min et al. [18] investigated an early intervention that started 1 day after surgery and consisted of 4 supervised exercise sessions and daily exercises at home during the first postoperative month. The programs were personalized and included stretching and muscle strength exercises to enhance the recovery of shoulder function. The intervention group fully recovered shoulder strength in the first month after surgery, and at six months, there was less loss of muscle mass, an increase in physical activity and an improvement in quality of life.

The importance of a multimodal nursing program based on WeChat in the early rehabilitation of women with breast cancer in the postoperative period to improve the quality of life of women postmastectomy was reported in two randomized clinical trials [20,21]. In addition to improvements in social/family and functional well-being, there was also a decrease in the fear of cancer recurrence [21].

A decrease in hospitalization time is one of the gains highlighted by the rehabilitation nurses. A shorter hospital stay reduces the negative impact of hospitalization on women undergoing a mastectomy if the continuity of care after surgery provides the necessary support in this transition process, empowers the women, and offers rehabilitation programs that contribute to their satisfaction and an improvement in quality of life [8].

These findings support the development of health policies and interventions for women with breast cancer during the postoperative period, within the scope of the multidisciplinary team. The care provided by rehabilitation nurses to women postmastectomy should be based on person-centered care, a preventive approach and professional development. Intervention strategies focused on supporting women and their families in the transition process, in addition to health education strategies, self-care training and the implementation of rehabilitation programs, which are determinants of the health gains stated by the participants. These findings may contribute to the design of undergraduate and graduate nursing curricula, as themes emerged that could be addressed in the training of new nurses and specialists in rehabilitation nursing.

This study has limitations related to the methodology, the intentional selection of participants and the data collection technique. The choice of a qualitative study, performed in a specific context, limits the transferability of the findings. The script of the narratives allows for some flexibility and is instrumental in the richness and depth of the findings, but it influences the diversity in the responses. The use of a researcher with experience in the qualitative analysis of the content of narratives and in the development of qualitative studies may have been important to minimize this risk of bias. Future studies with a larger number of participants should explore strategies that allow for the development of programs with pre-rehabilitation interventions for people diagnosed with breast cancer. This approach can be used by multidisciplinary teams and allows holistic care to be offered to the people involved, with the potential to improve outcomes throughout rehabilitation programs.

## 5. Conclusions

The findings of this study contribute to the understanding of the rehabilitation process of women who have had a mastectomy from the perspective of rehabilitation nurses. The rehabilitation of women postmastectomy involves a continuous multidisciplinary process, focusing on the restoration of the functionality, autonomy and quality of life of each woman. Mastectomy surgery, although essential in the treatment of breast cancer, has important impacts on the body image and functional capacity of women, affecting physical, emotional and social aspects.

The role of nurses specializing in rehabilitation nursing is crucial, as they provide technical and emotional support, promote self-care and prevent complications such as lymphedema and loss of mobility in the homolateral limb. The implementation of rehabilitation programs adapted to each woman, with interventions aimed at motor and cardiorespiratory training, as well as activities of daily living, is essential for full recovery.

Health education, combined with a humanized approach, allows women to face this transition process with greater resilience, minimizing the negative effects of surgery and promoting their reintegration into daily life.

## Figures and Tables

**Table 1:** Categories and indicators, 2025.

Category	Indicator
Meaning attributed to rehabilitation care for women who have had a mastectomy	− Development experience − Person-centered experience − Preventive experience
Intervention strategies	− Project development − Support in transition processes − Self-care training − Health education − Comfort care − Evaluation of functionality and health status − Implements motor and cardiorespiratory training programs − Implements ADL training program
Health gains arising from the implementation of care strategies	− Functional recovery − Autonomy − Acceptance of the health situation − Fewer complications − Improved quality of life − Shorter hospital stay

## Data Availability

The data will be available from Rogério Ferreira (ferrinho.ferreira@ipbeja.pt) upon reasonable request.

## Public Involvement Statement

No public involvement was had in any aspect of this research.

## Guidelines and Standard Statement

This manuscript was drafted against the standards for reporting qualitative research: a synthesis of recommendations (SQSR) for qualitative research. The complete list of reporting guidelines can be accessed via the equator network: https://www.equator-network.org/ (accessed on 3 March 2025).
